# Persistent inequalities in unplanned hospitalisation among colon cancer patients across critical phases of their care pathway, England, 2011–13

**DOI:** 10.1038/s41416-018-0170-2

**Published:** 2018-08-15

**Authors:** Camille Maringe, Bernard Rachet, Georgios Lyratzopoulos, Francisco Javier Rubio

**Affiliations:** 1 Cancer Survival Group, Department of Epidemiology and Population Health, London School of Hygiene and Tropical Medicine, London, WC1E 7HT UK; 2 Epidemiology of Cancer Healthcare and Outcomes (ECHO) Research Group, Department of Behavioural Science and Health, University College London, London, WC1E 7HB UK

**Keywords:** Epidemiology, Colon cancer, Health services

## Abstract

**Background:**

Reducing hospital emergency admissions is a key target for all modern health systems.

**Methods:**

We analysed colon cancer patients diagnosed in 2011–13 in England. We screened their individual Hospital Episode Statistics records in the 90 days pre-diagnosis, the 90 days post-diagnosis, and the 90 days pre-death (in the year following diagnosis), for the occurrence of hospital emergency admissions (HEAs).

**Results:**

Between a quarter and two thirds of patients experience HEA in the three 90-day periods examined: pre-diagnosis, post-diagnosis and before death. Patients with tumour stage I-III from more deprived backgrounds had higher proportions of HEAs than less deprived patients during all studied periods. This remains even after adjusting for differing distributions of risk factors such as age, sex, comorbidity and stage at diagnosis.

**CONCLUSIONS:**

Although in some cases HEAs might be unavoidable or even appropriate, the proportion of HEAs varies by socioeconomic status, even after controlling for the usual patient factors, suggestive of remediable causes of excess emergency healthcare utilisation in patients belonging to higher deprivation groups. Future inquiries should address the potential role of clinical complications, sub-optimal healthcare administration, premature discharge or a lack of social support as potential explanations for these patterns of inequality.

## Introduction

Hospital Emergency Admissions (or readmissions, HEAs) are generally defined as unplanned admissions to hospital. In the USA, rates of hospital emergency readmissions within 30 days are used as indicators of quality of care, and hospitals can be penalised if they present high rates of hospital readmissions.^1^ In England, the National Health Service monitors 90-days unplanned readmissions as an indicator of quality of care.^2^ HEAs have financial implications, as unplanned hospital admissions are more expensive than planned ones,^3^ and they utilise resources from the already pressured Accident and Emergency (A&E) department.^4^

In the context of cancer epidemiology in England, the literature has mainly focused on understanding the causes for HEAs within 90 days following colorectal cancer resection,^2^ describing the impact of deprivation on HEAs post-diagnosis in the Thames region,^5^ or analysing differential access to hospital care.^6,7^ Recently, great attention has also been paid to the analysis and impact of emergency presentations of cancer.^8,9^ However, there is insufficient evidence concerning the joint analysis of hospital admissions (HAs), elective or unplanned, of cancer patients in England for different periods in their trajectories. Despite wide-ranging causes for HEAs in different periods around the cancer diagnosis, examining different aspects of the cancer pathway jointly can reveal consistent patterns of variation for different patient groups. Large variations in cancer outcomes by deprivation remain unexplained by differential distribution of stage at diagnosis and access to treatment.^6,7,10^ Deprivation-related disparities in outcomes may be attributed to more complex variations in management and care, difficult to measure at population level. HEAs may be related to premature hospital discharge, overlooking the severity of certain symptoms, a lack of follow-up after the diagnosis and social support, or a delay in elective admission.

**Fig. 1:**
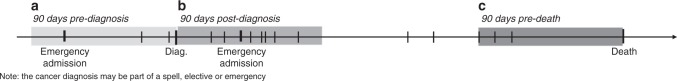
Diagram showing the different analysis periods. **a** 90 days pre-diagnosis, **b** 90 days post-diagnosis, and **c** 90 days pre-death (when death occurred in the 90 days to 1 year following diagnosis)

We present the analysis of HEAs in three key periods: 90 days pre-diagnosis, 90 days post-diagnosis and 90 days pre-death (Fig. 1). Depending on tumour factors, such as stage at diagnosis, these periods may be signs of (1) poor recognition of signs and symptoms by both patients and doctors; (2) poor quality discharge planning, poor social support, or a rapid evolution of the disease; and (3) the quality of life of terminal patients.

We report national proportions of HEAs for colon cancer patients in England. We also study the variation of HEAs by deprivation level. For both aims, we focus on the time period between three months before and a year after the date of diagnosis. To our knowledge, this is the first time the topic of emergency admission in colon cancer patients by deprivation is studied nation-wide and along the cancer care pathway.

## Methods

We analyse 65,020 records for patients diagnosed between 2011 and 2013 in England with a colon carcinoma (ICD-10 C18). These were linked to the Hospital Episode Statistics (HES) in-patients and A&E datasets that contain records of all hospital attendances between 2003 and 2014.

Information on patients’ age, deprivation, comorbidity and stage at diagnosis was available or derived^11,12^ from the cancer registrations linked to the National Bowel Cancer Audit Data (NBOCAT) and HES. Deprivation is measured at the Lower Super Output Area (LSOA) level (mean population 1500), using the Index for Multiple Deprivation (IMD) income domain.^13^ The IMD scores are ranked and split according to quintiles of their national distribution, thereby dividing the LSOAs in five groups of increasing deprivation. Patients are allocated to a deprivation group given their LSOA of residence at the time of diagnosis. Stage at diagnosis was classified using the Tumour, Node and Metastasis (TNM) staging system.^14^ Comorbidity information was derived from the HES data:^11^ A binary variable identifies comorbid patients. We checked the HES records in the 8 years preceding the colon cancer diagnosis for the occurrence of any of the 17 comorbidities that forms part of the Charlson score,^15^ or morbid obesity, given evidence that it may moderate decisions about surgical management and prognosis. For each patient, a diagnostic route, representing different types of care pathways to a diagnosis of cancer, was assigned by Public Health England using an algorithm based on linked data from cancer registration, Hospital Episodes Statistics, National Cancer Waiting Times and National Bowel Cancer Screening Programme.^16^

From the HES dataset, we defined unplanned admissions as: (i) any A&E admissions, and (ii) in-patient admissions where the method of admission (*admimeth* variable) was coded as “Emergency Admission, when admission is unpredictable and at short notice because of clinical need”.^17^ We looked at the admission status of the first or unique episode of a spell.^18^

We presented the analysis of HEAs in three key periods along the course of the disease: (1) 90 days pre-diagnosis, (2) 90 days post-diagnosis; and (3) 90 days pre-death (Fig. 1). Thus, the date of diagnosis as defined by the United Kingdom and Ireland Association of Cancer Registries (UKIACR) represents the reference point, around which we define pre- and post-diagnosis periods. In the 90 days pre-diagnosis, the 65,020 colon cancer patients could experience two possible outcomes: those diagnosed with cancer after one or more HEAs, and those diagnosed with cancer without any HEAs. In the 90 days post-diagnosis, these patients could be in one of four possible outcomes defined by the combination of their emergency admission status and vital status at 90 days. Analysing separately the patients who die before the end of the observation period is essential to avoid the phenomenon of “immortal bias”.^19^ We then selected patients who died between the 90th day and 12th month after diagnosis (*n* = 8681), and examined whether they had records of HEAs in the 90 days preceding death. Therefore, patients who had died within the first three months of diagnosis, or patients who were alive after a year did not form part of this sub-analysis.

Because of our prior interest in deprivation-related inequalities in HEAs, we presented proportions of HEAs by deprivation quintile. Since stage distribution varies by deprivation, and both stage and deprivation are associated with the occurrence of HEAs, we presented stratified analyses of HEAs by deprivation and stage at diagnosis (and route to diagnosis). The text often highlights differences between the two extreme deprivation groups (i.e. deprivation 1 and 5). The gradients, for the outcomes of interest, between all deprivation groups are given in tables and graphs.

In order to account further for differential distributions of risk factors between deprivation groups, we calculated indirectly standardised rates of emergency admissions for the most deprived patients in the 90 days following their colon cancer diagnosis. We applied the sex, age and comorbidity distribution of patients in the least deprived group, stratified by stage at diagnosis, to patients of the most deprived group. We can interpret the values as hypothetical proportions of HEA that would be observed in the most deprived category, had patients been subjected to the sex, age, and comorbidity allocation of least deprived patients. Given sex and age vary little by deprivation generally, it is a crude estimation of the role of comorbidity in the differential proportions of HEA between deprivation levels. We similarly calculated sex, age, comorbidity and stage-standardised rates restricted to patients with complete information on their stage at diagnosis.

## Results

### Inequalities in HEA by patient factors

#### Pre-diagnosis

**Fig. 2:**
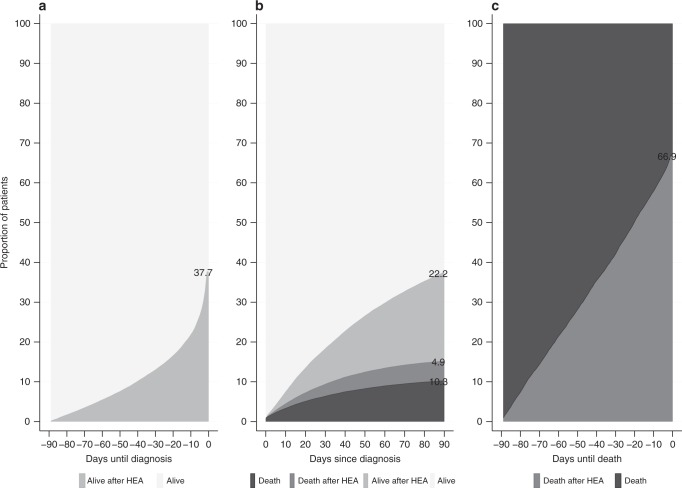
Time-varying proportions of HEA in the 90 days pre-diagnosis (**a**), 90 days post-diagnosis (**b**), and 90 days pre-death (**c**)

In the 90 days preceding their colon cancer diagnosis, over a third (38%) of patients experienced at least one HEA (Table 1). Over half of the first of these pre-diagnosis HEAs occurred in the 30 days prior to diagnosis (Fig. 2a).

**Table 1:** Distribution of the outcomes by patient characteristics, within each period, of the 65,020 patients diagnosed with colon cancer in England in 2011–13

	90-day pre-diagnosis				90-day post-diagnosis								90-day pre-death (*N* = 8681)			
	Alive		Alive after HEA		Alive		Alive after HEA		Death		Death after HEA		Death		Death after HEA	
	*N*	%	*N*	%	*N*	%	*N*	%	*N*	%	*N*	%	*N*	%	*N*	%
Total	40,498	62.3	24,522	37.7	40,688	62.6	14,441	22.2	6689	10.3	3202	4.9	2872	33.1	5809	66.9
Age at diagnosis (mean age)	71.7		72.7		71.2		70.1		80.0		75.5		78.6		74.5	
Sex																
F	18,598	60.2	12,301	39.8	19,078	61.7	6803	22.0	3526	11.4	1492	4.8	1522	35.5	2768	64.5
M	21,900	64.2	12,221	35.8	21,610	63.3	7638	22.4	3163	9.3	1710	5.0	1350	30.7	3041	69.3
*p*-value				<0.001^a^								<0.001^b^				< 0.001^a^
Deprivation																
1	9602	67.3	4670	32.7	9412	65.9	3018	21.1	1222	8.6	620	4.3	635	35.5	1153	64.5
2	9342	65.0	5039	35.0	9282	64.5	3065	21.3	1360	9.5	674	4.7	649	34.7	1223	65.3
3	8604	62.8	5087	37.2	8669	63.3	2923	21.3	1439	10.5	660	4.8	605	33.3	1214	66.7
4	7553	59.2	5209	40.8	7661	60.0	2955	23.2	1457	11.4	689	5.4	581	32.4	1210	67.6
5	5397	54.4	4517	45.6	5664	57.1	2480	25.0	1211	12.2	559	5.6	402	28.5	1009	71.5
*p*-value		0.001^c^		0.001^c^		0.002^c^		0.018^c^		<0.001^c^		0.001^c^		0.008^c^		0.008^c^
Stage at diagnosis																
1	5550	81.9	1230	18.1	5467	80.6	1176	17.3	88	1.3	49	0.7	76	37.3	128	62.7
2	9708	65.3	5160	34.7	10,846	72.9	3257	21.9	578	3.9	187	1.3	222	30.8	499	69.2
3	8479	63.8	4808	36.2	9176	69.1	3301	24.8	557	4.2	253	1.9	364	28.2	927	71.8
4	8111	52.8	7247	47.2	6681	43.5	3885	25.3	2876	18.7	1916	12.5	1407	32.5	2925	67.5
*p*-value		0.023^c^		0.023^c^		0.038^c^		0.027^c^		0.071^c^		0.087^c^		0.214^c^		0.214^c^
Missing stage	8650	58.7	6077	41.3	8518	57.8	2822	19.2	2590	17.6	797	5.4	803	37.6	1330	62.4
Comorbidity																
0	26,474	69.3	11,752	30.7	25,587	66.9	8362	21.9	2666	7.0	1611	4.2	1443	32.8	2960	67.2
1	14,024	52.3	12,770	47.7	15,101	56.4	6079	22.7	4023	15.0	1591	5.9	1429	33.4	2849	66.6
*p*-value				<0.001^a^								<0.001^b^				0.538^a^

^a^Fisher’s test for count data. Tests for the equality of two binomial populations. The test was performed with the R command fisher.test()

^b^Chi-square test. Tests for the equality of two multinomial populations. The test was performed with the R command chisq.test()

^c^Correlation test for trend: Test for a positive or negative correlation of the means. The *p*-values correspond to one-sided tests. The *p*-value is obtained with the R command cor.test()

These HEA events were somewhat more frequent among women (40% vs 36% in men, *p* < 0.001), and substantially more frequent among patients subsequently diagnosed at more advanced stage (47% vs 18% for patients diagnosed at stages IV and I, respectively, *p* = 0.023), those with increasing deprivation (46% vs 33% in least and most deprived patients, respectively, *p* = 0.001) and those with at least one comorbidity (48% vs 31% among those without, *p* < 0.001, Table 1).

#### Post-diagnosis

Up to 15% of patients died in the 90 days following their colon cancer diagnosis, including 5% after at least one HEA. There are marked differences by stage at diagnosis, with increasing proportions of patients who died with or without HEA with increasing stage (Table 1). Patients who died within the first 90 days after their diagnosis were 78.6 years compared to 71 years for those who survived that period. There is some variation in the proportions of patients dying, with (*p* = 0.001) or without (*p* < 0.001) HEA, by deprivation.

Among the majority of patients who survived to 3 months, HEAs were observed in 22%. This proportion varied little by sex, and comorbidity, but was substantially greater in more deprived patients (*p* = 0.018) and those diagnosed in advanced stage (*p* = 0.027, Table 1). Overall, there were higher proportions of patients without comorbidities (67%), stage I disease (81%), and least deprived (66%) who did not experience any HEA during the 3 months post-diagnosis than those patients with comorbidities (56%), stage IV disease (44%) or most deprived (57%) (Table 1).

There was a sharp increase in proportions of patients dying (with or without HEA) in the first days after diagnosis, and that increase flattened out after 20–30 days after diagnosis. On the contrary, the proportions of HEAs kept increasing through to 90 days after diagnosis (Fig. 2b).

#### Pre-death

Among the 8,681 patients who died between 90 days and a year of their colon cancer diagnosis, on average 67% experienced at least one HEA in the 90 days prior to their death (Table 1 and Fig. 2c). Such HEA events were more common among male (69% vs 65% in female, *p* < 0.001) and more deprived (72% to 65% from most to least deprived, *p* = 0.008) fatal cases. There was a constant increase in the proportion of patients with HEA with increasing time to death (Fig. 2c).

Missing information on stage was affecting between 20% (alive patients with a HEA after diagnosis) and 39% (patients who died without HEA in the 90 days after diagnosis) of patients.

### Diagnostic route

Diagnostic route was linked to patterns of HEAs pre-diagnosis and the proportions of HEAs post-diagnosis and pre-death, when death happened in the 90 days to first year after the diagnosis (Annex 1). Patients diagnosed through screening had lowest proportions of HEAs at all times (<5%), in contrast to patients diagnosed via Emergency, which showed higher proportions of HEAs or death. In the 90 days pre-diagnosis, patients diagnosed through the two-week-wait had low proportions of HEAs (5–10%). In the 90 days post diagnosis, patients diagnosed following GP referral, in-patient elective, other outpatient and two-week-wait had lower proportions of death (with or without HEAs, 10% or less), but similar proportions of patients with one or more HEAs to patients diagnosed through emergency or unknown routes (~20%), see Annex 1.

### Stage-specific deprivation-related inequalities in HEA

**Fig. 3:**
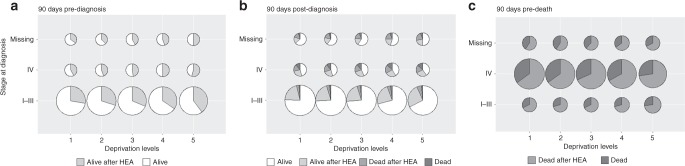
Distribution of each outcome, by period, deprivation quintile and stage at diagnosis: **a** 90 days pre-diagnosis, **b** 90 days post-diagnosis, **c** 90 days pre-death

Around the time of diagnosis, there were linearly increasing proportions of patients with adverse outcomes (HEAs pre- and post-diagnosis and death within 90 days of diagnosis, Annex 2A&B) with increasing deprivation (Fig. 3a, b), at all stages at diagnosis. The discrepancy in adverse outcomes between deprivation groups for patients dying in their first year was less striking at stages I–III, but still evident for patients diagnosed at stage IV or missing stage (Fig. 3c).

Before the diagnosis, the proportions of patients with at least one HEA differed between least and most deprived (Table 2): respectively 28% and 40% of stage I–III patients, 42% and 55% of stage IV patients, and 36% and 50% of missing stage patients had HEAs before their cancer diagnosis. Those differences by deprivation remained in the 90 days after diagnosis for patients diagnosed at stages I–III or with no valid stage recorded. Patients diagnosed at stage IV did not show striking variations by deprivation in the proportions of each of the four outcomes in the 90 days following diagnosis and patterns through time were similar across deprivation groups. In the 90 days pre-death, most deprived patients had between 5% and 7% higher proportions of death within 90 days of one or more HEAs than least deprived patients.

**Table 2:** Proportions of each outcome (with 95% CI), 90 days after diagnosis, for least and most deprived patients. Indirectly standardised proportions^a^ for most deprived patients

		Deprivation		Standardised proportions (most deprived)		
Outcome	Stage	Least	Most	Age, sex	Age, sex, comorbidity	Age, sex, comorb., stage (Complete case)^b^
*a. 90 days pre diagnosis*						
Proportion with HEA						
	I–III	27.7%	39.7%	39.2%	37.0%	
	95% CI	26.7%;28.7%	38.3%;41.0%	37.9%;40.5%	35.7%;38.3%	
	IV	41.8%	54.5%	54.2%	51.7%	
	95% CI	40.1%;43.5%	52.5%;56.5%	52.2%;56.2%	49.7%;53.7%	
	Missing	35.6%	49.9%	48.7%	46.4%	
	95% CI	34.0%;37.3%	47.8%;51.9%	46.6%;50.7%	44.4%;48.5%	
	All (complete case)^b^	31.9%	44.3%			41.3%
	95% CI	31.0%;32.8%	43.2%;45.4%			40.2%;42.4%
*b. 90 days post diagnosis*						
Dead						
	I–III	2.7%	4.7%	5.0%	4.4%	
	95% CI	2.4%;3.1%	4.1%;5.3%	4.4%;5.6%	3.9%;5.0%	
	IV	16.7%	19.0%	20.0%	19.4%	
	95% CI	15.4%;18.0%	17.4%;20.6%	18.3%;21.6%	17.8%;21.0%	
	Missing	14.5%	22.4%	22.1%	21.5%	
	95% CI	13.2%;15.7%	20.7%;24.1%	20.4%;23.8%	19.8%;23.2%	
	All (complete case)^b^	6.9%	9.2%			8.9%
	95% CI	6.4%;7.4%	8.5%;9.8%			8.2%;0.0%
Proportion with HEA						
Dead						
	I–III	1.1%	1.8%	1.9%	1.8%	
	95% CI	0.9%;1.3%	1.4%;2.1%	1.5%;2.2%	1.4%;2.1%	
	IV	11.9%	13.9%	14.1%	13.8%	
	95% CI	10.8%;13.0%	12.5%;15.2%	12.7%;15.6%	12.4%;15.2%	
	Missing	4.5%	6.0%	6.0%	5.8%	
	95% CI	3.7%;5.2%	5.0%;7.0%	5.0%;6.9%	4.8%;6.7%	
	All (complete case)^b^	4.3%	5.5%			5.3%
	95% CI	3.9%;4.7%	5.0%;6.0%			4.8%;5.8%
Alive						
	I–III	20.7%	26.0%	25.6%	24.9%	
	95% CI	19.8%;21.6%	24.8%;27.2%	24.4%;26.8%	23.7%;26.1%	
	IV	25.7%	26.5%	25.4%	25.4%	
	95% CI	24.2%;27.2%	24.8%;28.3%	23.6%;27.1%	23.6%;27.1%	
	Missing	17.6%	21.1%	21.3%	20.8%	
	95% CI	16.3%;18.9%	19.4%;22.8%	19.6%;22.9%	19.1%;22.5%	
	All (complete case)^b^	22.1%	26.2%			25.1%
	95% CI	21.4%;22.9%	25.2%;27.2%			24.1%;26.0%
*c. 90 days pre death*						
Proportion with HEA						
	I–III	68.2%	73.1%	72.3%	71.5%	
	95% CI	63.8%;72.6%	68.8%;77.4%	67.9%;76.6%	67.1%;75.9%	
	IV	65.1%	73.0%	71.4%	70.7%	
	95% CI	62.0%;68.2%	69.6%;76.3%	67.9%;74.8%	67.2%;74.1%	
	Missing	59.0%	66.9%	66.9%	65.0%	
	95% CI	54.3%;63.6%	61.9%;71.8%	61.9%;71.8%	59.9%;70.0%	
	All (complete case)^b^	66.1%	73.0%			70.9%
	95% CI	63.6%;68.6%	70.3%;75.7%			68.2%;73.7%

^a^Indirect standardisation of proportions for most deprived patients use the age and sex, age, sex and comorbidity by stage, and age, sex, comorbidity and stage distributions observed in least deprived patients.

^b^Complete cases are patients with a valid record of stage

Considering observed non-standardised proportions, it is apparent that overall most deprived patients had higher proportions of all types of HEA’s events studied (across all three 90-day periods of interest), particularly for patients in stages I–III (Table 2). In these groups, proportions of adverse outcomes remained higher in most deprived compared to least deprived patients, even after removing the impact that differential distributions of sex, age, and comorbidity (and stage) might have on those proportions.

Patients diagnosed at stage IV had slightly overlapping 95% confidence intervals around their proportions of outcomes in the 90 days following diagnosis, suggesting a milder deprivation gap in this sub-group.

Indirect standardisation, i.e. applying the distributions of age, sex, comorbidity and stage observed in the least deprived patients to the most deprived patients, hardly changed the proportions of any of the outcomes studied in all three time-periods among the most deprived patients. That is, the most deprived group of patients experience higher proportions of emergency healthcare than the least deprived group.

In all three phases of the care pathway studied, both the least and the most deprived groups showed similar increasing trends with time in their proportions of each outcome (Annex 2). Trends in elective admissions were identical in least and most deprived patients (Annex 3).

## Discussion

In the 90 days before the diagnosis, over a third of patients experience at least one HEA, in line with the reported proportions of patients diagnosed via emergency presentation within 28 days of diagnosis.^16^ In total 85% of the patients survived the 90 days post-diagnosis and a quarter of them experienced at least one HEA in this period. By contrast, among the remaining 15% who died, a third (i.e. 5% of all patients) experienced at least one HEA in this post-diagnosis period. The proportion of patients who experienced at least one HEA rose to two thirds in the 90 days pre-death, among those who died in the first year after diagnosis. Our study highlights a clear deprivation-related variability for patients diagnosed at stages I–III: patients from more deprived background exhibit higher rates of HEAs than least deprived patients (with a decreasing gradient for intermediate deprivation levels) pre- and post-diagnosis. This phenomenon is not explained by differences in the distribution of age, sex, presence of comorbidity, and stage of disease. This is in line with the results found in the US^20^ and in the England Thames region.^5^ In contrast, we did not find large differences in the proportions of elective admissions across deprivation groups in the 90 days following diagnosis. Stage IV patients exhibit less marked deprivation-related variability in terms of HEAs, which may be related to the terminal status of those patients.

These analyses are based on all colon cancer patients diagnosed in England in the years 2011–13. The patients’ records were linked to the A&E and in-patient hospital data. Any differences between groups of patients highlighted here are not likely due to chance given the population-based nature of the data. We defined the emergency status of a spell in HES^18^ based on the first or unique HES episode of that spell, i.e. the emergency status of further episodes of a spell are not considered.

Another contribution of this work is that it provides appropriate descriptive tools for HEA data. More specifically, given that in the 90 days after the diagnosis some patients die, it implies that, for those patients, the events of interest (HEAs) cannot occur in a certain period during the follow-up time, i.e. in the time following death. This phenomenon is known as immortal bias^19^ and needs to be taken into account in order to avoid drawing biased conclusions.

A limitation of our analysis is that it excludes elective and emergency admissions to private hospitals. This may induce a slight under estimation of the number of admissions in the least deprived group, which is the most likely group to attend private hospitals. Nevertheless, we do not expect the inclusion of such admissions to private hospitals to affect the conclusions presented in this paper.^21^

This study highlights consistent patterns of increased proportions of HEA with increasing deprivation among the colon cancer patients at stages I–III. Similar patterns have been reported in the general population.^22^ These are stable throughout the diagnosis and treatment pathways. Furthermore, we emphasise that there are differences between deprivation groups before the diagnosis, i.e. when patients are still considered part of the general population.

Interestingly, once patients are diagnosed, and should therefore undertake the same scrutinised treatment and follow-up for their disease, the difference between deprivation groups remains, even after the influence of case-mix factors is taken into account. This is suggestive of differential management as well as the potential contribution of different socio-cultural factors including use of hospital and palliative care, family and social support, and hospital as a place of death. Evidence of this may be found in late-stage patients dying in the year following their colon cancer diagnosis, whereby most healthcare need can be assumed to relate to palliative care and support. Least deprived patients show, perhaps unavoidable, high proportions of HEAs in the 90 days pre-death (65%), but up to 72% of most deprived patients visit A&E prior to death—the difference in these percentages can provide a measure of potentially avoidable HEAs in the most deprived group.

Comorbidity information was captured from linkage of the HES records to the cancer registration dataset for the years preceding diagnosis.^11^ Comorbidities derived using HES diagnostic codes provide a valid assessment of the health status of patients.^23,24^ An 8-year period before the colon cancer diagnosis was used to classify comorbid patients. This represents the largest assessment period available for patients diagnosed in 2011, and therefore minimises misclassification of patients into the “not-comorbid” category when in fact they are comorbid.

These results point out that there is an excess of potentially avoidable HEAs in the more deprived groups compared to the least deprived group. In order to reduce HEAs, interventions should focus on deprived groups and aim at integrating social and health care. For instance, patients that exhibit recurrent HEAs in a short period of time reflect either clinical complications, sub-optimal healthcare administration, premature discharge or a lack of social support. Disentangling the causes for recurrent HEAs requires a case by case analysis, and an efficient communication with the health professionals.

The aim of these analyses is to be paving the way for a more systematic study of hospital emergency admissions for cancer patients in different periods along their diagnosis pathways. Although we chiefly focus on deprivation-related inequalities in hospital admissions, studying other types of inequalities is also relevant. For instance, the factors (clinical or managerial) behind the observed differences in HEAs between male and female patients in the pre-diagnosis period deserve further investigation.^25^ Nonetheless, a lot of information is available in the hospital data, such as admission codes, length of stay, details of the consultant caring for the patients, and cost of the hospital admissions. Identifying, extracting and synthesising further data items is key to understand better the differences in emergency access to healthcare around a colon cancer diagnosis, identify and characterise sub-optimal pathways.

In the different periods, the trajectories of cancer patients can be of very different nature due to wide-ranging causes. A lack of cancer symptoms awareness (patient-level) and sub-optimal management, including delay in the medical attention or premature discharge (system-level) can affect the occurrence and recurrence of HEAs.

In the 90 days before the diagnosis, there are opportunities for a timely diagnosis and earlier detection of people with signs and symptoms suggestive of cancer.^26^ In the 90 days after the diagnosis, reducing HEAs may help provide optimal healthcare and treatment assignment. Receiving healthcare attention in HEAs is far from being the optimal healthcare pathway. Looking at patients who die within a year, 80% of them had an elective admission within 90 days of their death, happening, on average, around two months before death. However, roughly twice the proportion of most deprived patients die after a HEA, with no elective admissions, compared to the least deprived group. Initiatives to allow patients to decide where to be cared for and where to die are already in place (e.g. The Marie Curie Cancer Care Delivering Choice Programme).^27^ However, given the differences in HEAs by deprivation that we emphasise, it is important to make sure that these initiatives are being made available equally to all patients, regardless of their socioeconomic background, and that the patients’ choice is well informed.

Given the financial and prognostic implications of HEAs, it is important to understand the patterns of hospital admissions of cancer patients. Identifying the characteristics of patients associated with higher proportions of HEAs helps targeting campaigns and policies on vulnerable sectors. Our study identifies cancer patient groups at higher risk of emergency admissions, which can be considered by UK healthcare providers to plan consultation rates, follow-up strategies, comorbidity management, and social support to help reduce the rate of HEAs.

## Electronic supplementary material

Appendix 1

Appendix 2

Appendix 3
