# Supplementary figures and images for: Persistent inequalities in unplanned hospitalisation among colon cancer patients across critical phases of their care pathway, England, 2011–13
Source: Br J Cancer. 2018 Aug 15;119(5):551–7. doi: 10.1038/s41416-018-0170-2 (PMC6162238; doi:10.1038/s41416-018-0170-2)

ANNEX 1. Proportions of each outcome by the end of each period, by route to diagnosis

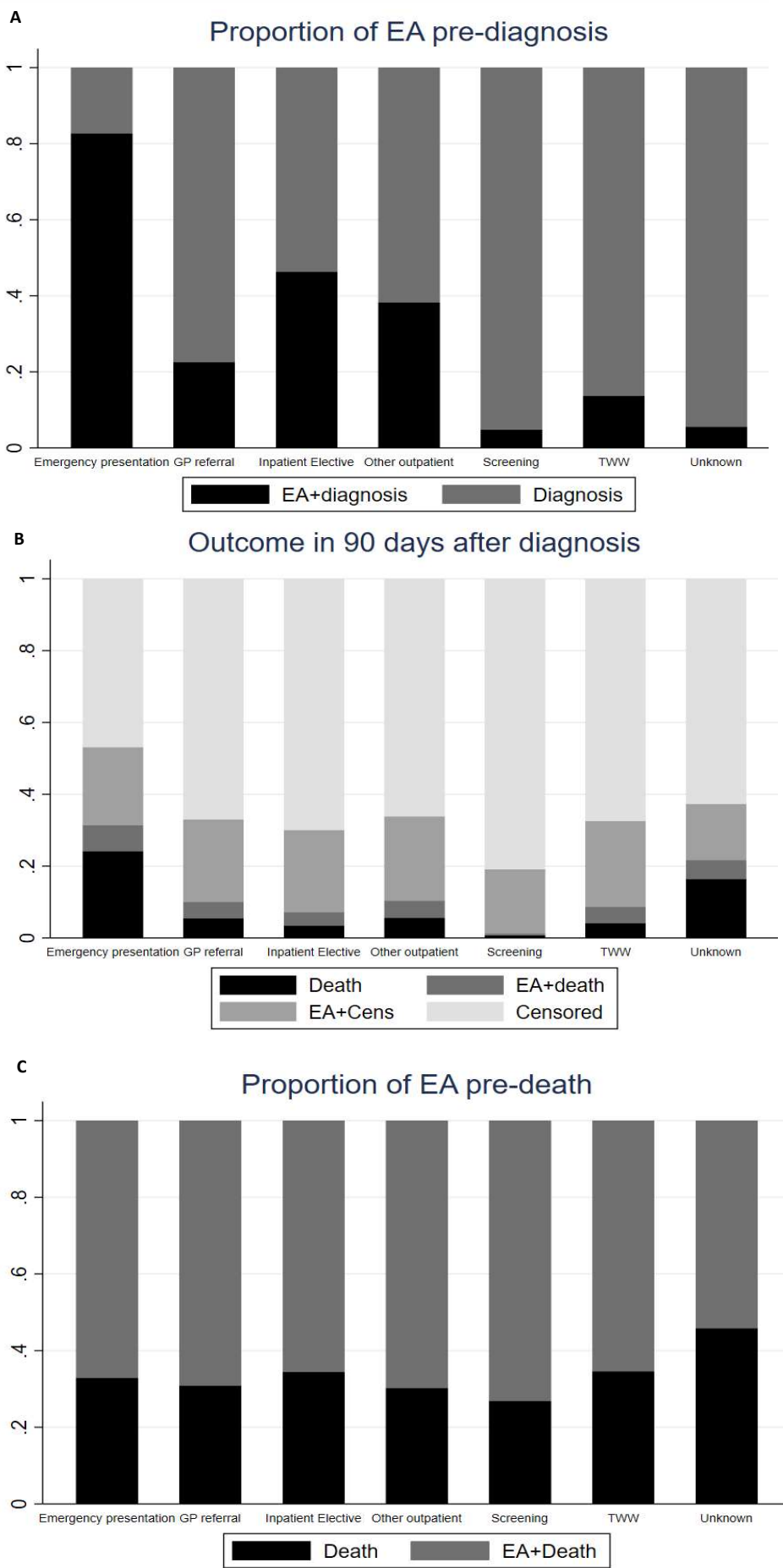

Supplement: Supplementary file 1 — Appendix 1 [file 41416_2018_170_MOESM1_ESM.pdf]
