# Supplementary material for: Persistent inequalities in unplanned hospitalisation among colon cancer patients across critical phases of their care pathway, England, 2011–13
Source: Br J Cancer. 2018 Aug 15;119(5):551–7. doi: 10.1038/s41416-018-0170-2 (PMC6162238; doi:10.1038/s41416-018-0170-2)
Supplement: Supplementary file 3 — Appendix 3 [file 41416_2018_170_MOESM3_ESM.pdf]

ANNEX 3. Time-varying proportions of elective admissions in the 90 days pre diagnosis (A), 90 days post diagnosis (B) and 90 days pre death[1](C), by stage for least and most deprived patients

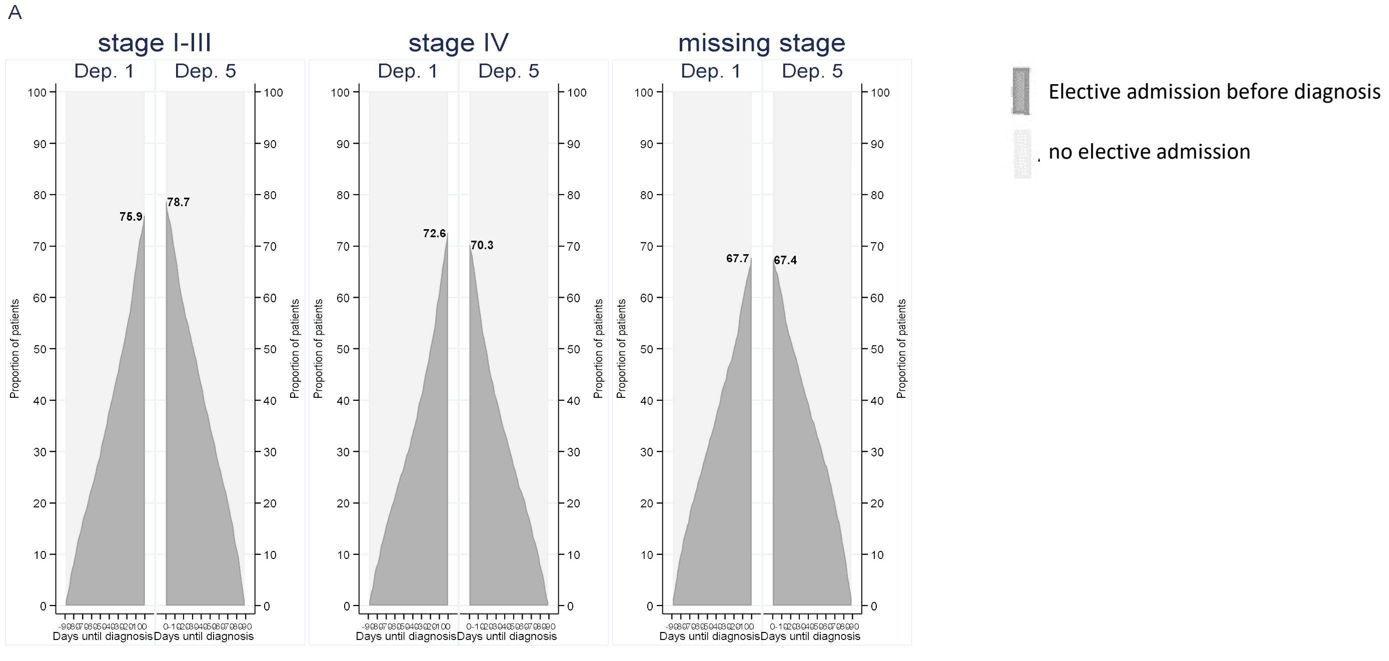

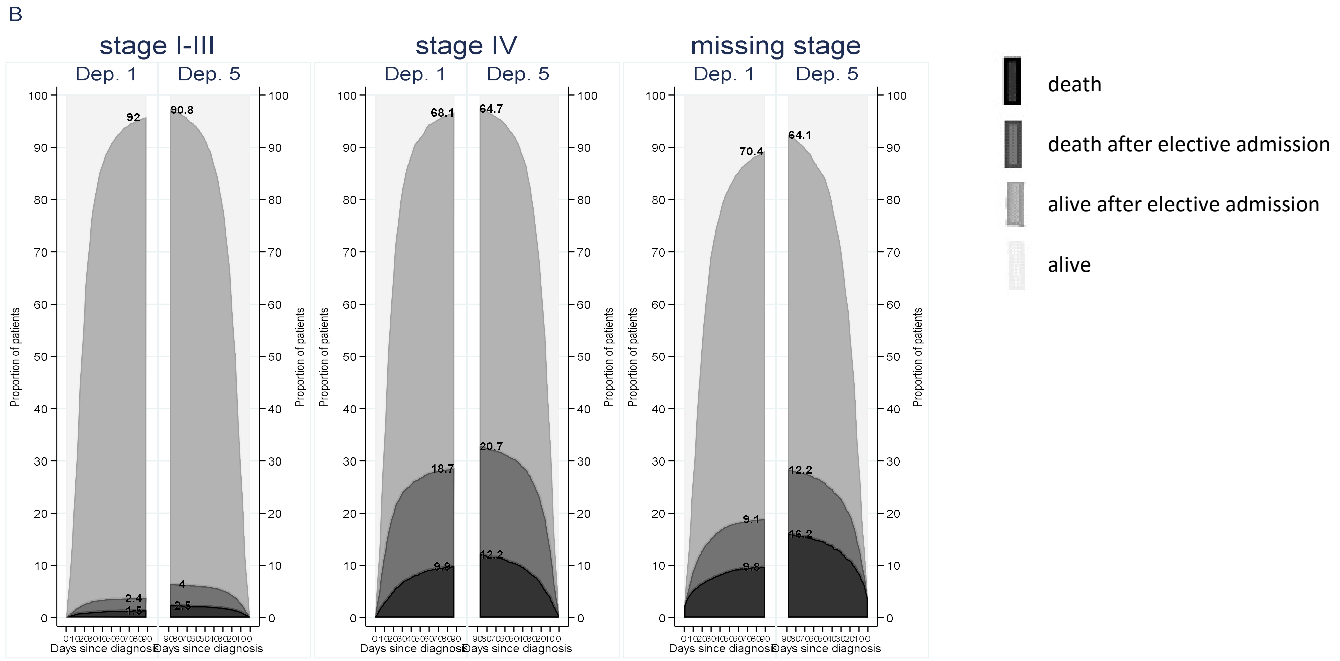

[1] for patients who die between 3 and 12 months after their diagnosis

dep 1: least deprived

dep 5: most deprived

ANNEX 3. Time-varying proportions of elective admissions in the 90 days pre diagnosis (A), 90 days post diagnosis (B) and 90 days pre death[1](C), by stage for least and most deprived patients

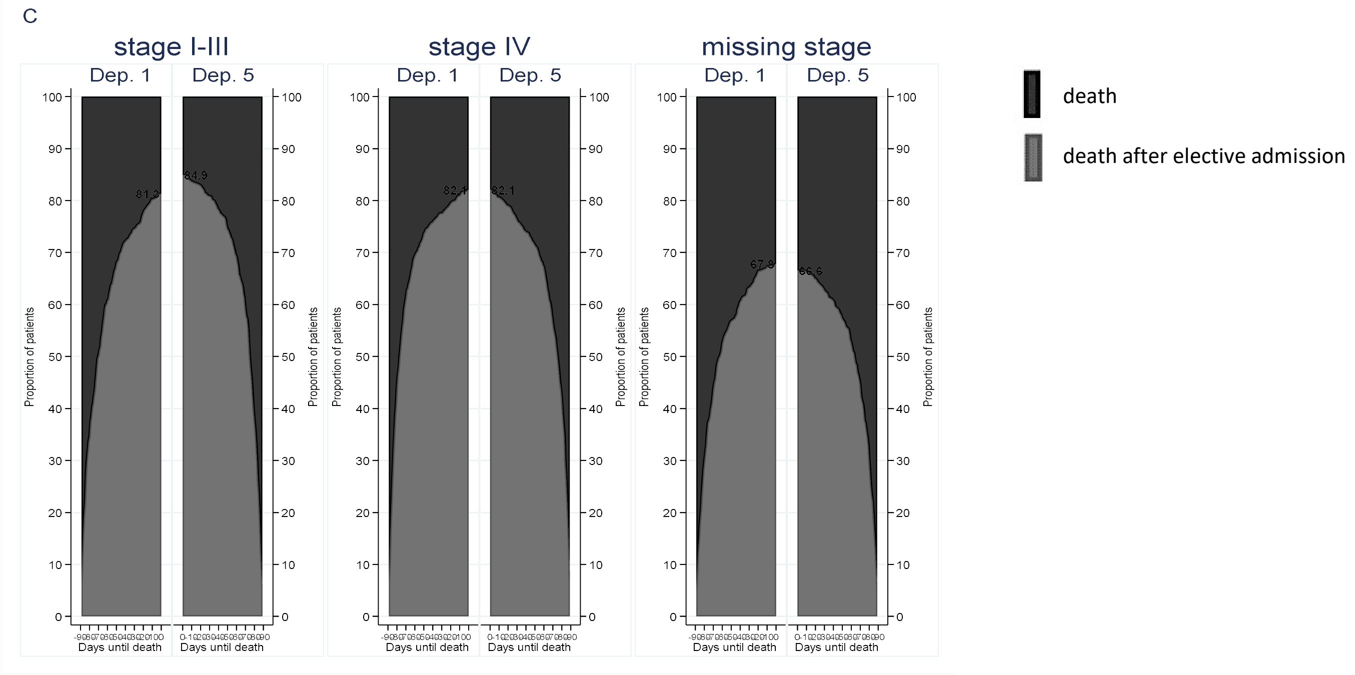

[1] for patients who die between 3 and 12 months after their diagnosis
dep 1: least deprived
dep 5: most deprived
